# TCR-independent functions of Th17 cells mediated by the synergistic actions of cytokines of the IL-12 and IL-1 families

**DOI:** 10.1371/journal.pone.0186351

**Published:** 2017-10-12

**Authors:** Yun Kyung Lee, Ashley E. Landuyt, Stefani Lobionda, Panida Sittipo, Qing Zhao, Craig L. Maynard

**Affiliations:** 1 Department of Pathology, University of Alabama at Birmingham, Birmingham, AL, United States of America; 2 Soonchunhyang Institute of Medi-Bioscience (SIMS), Soonchunhyang University, Cheonan-si, Korea; 3 Department of Medicine, University of Alabama at Birmingham, Birmingham, AL, United States of America; Ohio State University, UNITED STATES

## Abstract

The development of Th17 cells is accompanied by the acquisition of responsiveness to both IL-12 and IL-23, cytokines with established roles in the development and/or function of Th1 and Th17 cells, respectively. IL-12 signaling promotes antigen-dependent Th1 differentiation but, in combination with IL-18, allows the antigen-independent perpetuation of Th1 responses. On the other hand, while IL-23 is dispensable for initial commitment to the Th17 lineage, it promotes the pathogenic function of the Th17 cells. In this study, we have examined the overlap between Th1 and Th17 cells in their responsiveness to common pro-inflammatory cytokines and how this affects the antigen-independent cytokine responses of Th17 cells. We found that in addition to the IL-1 receptor, developing Th17 cells also up-regulate the IL-18 receptor. Consequently, in the presence of IL-1β or IL-18, and in the absence of TCR activation, Th17 cells produce Th17 lineage cytokines in a STAT3-dependent manner when stimulated with IL-23, and IFN© via a STAT4-dependent mechanism when stimulated with IL-12. Thus, building on previous findings of antigen-induced plasticity of Th17 cells, our results indicate that this potential of Th17 cells extends to their cytokine-dependent antigen-independent responses. Collectively, our data suggest a model whereby signaling via either IL-1β or IL-18 allows for bystander responses of Th17 cells to pathogens or pathogen products that differentially activate innate cell production of IL-12 or IL-23.

## Introduction

Th17 cells are characterized by the production of IL-17A, IL-17F and IL-22 and perform diverse roles in tissue immunity. Th17 cells are induced by the combined actions of TGFβ and IL-6 on TCR-activated naïve CD4 T cells which promote expression of the lineage-associated transcription factors, RORγt [1], RORα [2] and IRF4 [3]. IL-6, as well as IL-21 –an autocrine factor induced by IL-6, promote up-regulation of the IL-23 receptor (IL-23R) [4, 5] in parallel to IFNγ-induced up-regulation of the IL-12Rβ2 on Th1 cells [6–8]. The up-regulation of IL-23R facilitates the role for IL-23 in the effector functions of committed Th17 cells. Indeed, IL-23 signaling in Th17 cells was shown to be crucial for the pathogenic ability of this lineage in an animal model of multiple sclerosis [9]. Th17 cell differentiation is also accompanied by up-regulation of IL-12Rβ1 and IL-12Rβ2, which render these cells responsive to IL-12 [10]. We, and others have demonstrated that responsiveness to IL-12 results in the acquisition of a Th1-like phenotype by developing Th17 cells [10, 11].

In addition to the aforementioned cytokines, members of the IL-1 family of cytokines have been linked to Th17 differentiation and/or function. Specifically, IL-1 amplifies Th17 differentiation [12–14] and, in co-operation with IL-6 and IL-23 induces diversion of Foxp3+ iTreg precursors to the Th17 lineage [15]. IL-1 function is also critical in Th17-related animal models of autoimmune disease such as experimental autoimmune encephalomyelitis (EAE) [16] and spontaneous arthritis [17].

In the presence of a STAT3 activator, IL-1β can induce antigen-independent cytokine production by Th17 cells and another IL-1 family member, IL-33, in combination with a STAT5 activator induces TCR-independent secretion of IL-13 by Th2 effectors [18]. Thus, adding these two cascades to the previously described collaboration between IL-12 and IL-18 in inducing production of IFNγ by Th1 effectors [19, 20], it was postulated that committed effector CD4 T cells retain the capacity to function independent of continuous TCR activation provided they received synergistic signals from a STAT activator and an IL-1 family cytokine; IL-18 for Th1 cells, IL-33 for Th2 cells, and IL-1 for Th17 cells. Among effector T cells, expression of IL-33R and IL-1R are restricted to the Th2 and Th17 lineages respectively [18, 21]. However, IL-18R is expressed by both Th1 and Th17 cells, albeit at a reduced level on the latter [22]. Yet, unlike for Th1 cells, there is no documented role for IL-18 in TCR-independent functions of Th17 cells. Also, because Th17 cells express every receptor necessary to confer responsiveness to both IL-12 and IL-23 [23], it is conceivable that these cytokines, acting in concert with IL-1 and IL-18 can regulate the balance between the divergent fates of committed Th17 cells.

We performed experiments to compare the impact of Th17 cell responsiveness to IL-18, particularly in the presence of IL-12 or IL-23. In addition, we examined the effect of co-operation between these STAT-activating cytokines (IL-12 or IL-23) and IL-1 or IL-18, on TCR-independent cytokine production by committed Th17 cells. Considering our earlier observation that cells committed to the Th17 lineage can revert to Th1-like IFNγ-producing cells when reactivated with antigen in the presence of IL-12 [10], we were interested in whether the observed Th17 plasticity can be induced following cytokine-dependent but antigen-independent activation. Here we report that differentiated Th17 cells respond to IL-1β or IL-18 in concert with IL-23 to produce Th17-associated cytokines. The ability to respond to these stimuli is imprinted during initial differentiation of Th17 from naïve T cells and is enhanced when Th17 cells are induced in the presence of exogenous IL-23. Moreover, together with IL-1β or IL-18, IL-12 induces production of IFNγ by Th17 cells, similar to the previously reported plasticity of antigen-stimulated Th17 cells. Collectively, our data demonstrate that Th17 differentiation induces a transcriptional profile that ensures continued production of key functional molecules in the absence of further direct antigenic exposure and also imprints the potential to transition to Th1-like IFNγ-producing cells in response to appropriate stimuli.

## Materials and methods

### Mice

The following mice were purchased from the Jackson Laboratories and/or bred at the University of Alabama at Birmingham (UAB): C57BL/6J (B6), B6.OT-II TCR transgenic mice (OT-II), BALB/cByJ (BALB/c), B6.129S1-*Il12b*^*tm/Jm*^/J (*Il12b*^-/-^ or *Il12p40*^-/-^), 129S2-Stat4^tm1Gru^/J (*Stat4*^-/-^) and B6.*Il10*^-/-^. The generation of *IL-17F*/*Thy1*.*1* knock-in mice was described previously [10]. All animals were bred and maintained in accordance with UAB Institutional Animal Care and Use Committee (IACUC) regulations.

### CD4 T cell isolation and culture

CD4 T cells were purified from pooled spleen and lymph nodes using Dynabeads mouse CD4 beads followed by DETACHaBEAD according to manufacturer’s instructions (Invitrogen). The naïve (CD25^lo^CD62L^hi^) fraction was enriched by FACS sorting. Unless otherwise indicated, naïve cells were cultured at a ratio of 1:5 with irradiated splenic feeder cells for 7 days in RPMI containing 10% FBS, 100 IU/ml penicillin, 100 μg/ml streptomycin, 1 μM sodium pyruvate, 1x non-essential amino acids, 2.5 μM β-mercaptoethanol, 2 μM L-glutamine (R-10). OT-II TCR transgenic CD4^+^ cells were activated with 5 μg/ml ovalbumin peptide (OVAp), whereas non-transgenic cells were stimulated with 2.5 μg/ml anti-CD3 (clone 145-2C11). Th1-polarizing conditions: IL-12 (10ng/ml) and anti-IL-4 mAb (clone 11B11, 10 μg/ml). Th17-polarizing conditions: 5 ng/ml rh TGFβ1 (R&D Systems), 20 ng/ml rmIL-6 (R&D Systems), 10 μg/ml anti-IFNγ mAb (clone XMG1.2) and 10 μg/ml anti-IL-4 mAb in the absence or presence of 10 ng/ml IL-23 (R&D Systems). For repetitive polarization, viable T cells were recovered on day 7 and activated with fresh splenic feeders, and the same cytokines and antibody mixtures as initial culture condition.

### Flow cytometric analysis

CD4 T cells were collected and where indicated, stimulated with PMA (50 ng/ml; Sigma) and ionomycin (750 ng/ml; Calbiochem) for 5 hours in the presence of Golgi Plug (brefeldin A, BD biosciences) or with cytokines only for 12 hours, with GolgiPlug added for the last 5 hours. Intracellular staining was performed as previously described [10]. LIVE/DEAD Fixable Green Dead Cell Stain (Invitrogen) was used extracellularly to exclude dead cells in flow cytometric analyses. Phycoerythrin (PE)-conjugated anti-CD90.1 (OX-7) and anti-IL-17A (TC11-18H10) were purchased from BD Biosciences; allophycocyanin (APC)-conjugated anti- IFNγ (XMG1.2), PE-Cy7-conjugated anti-CD4 (GK1.5), and PE-conjugated anti-IL-18Rα (P3TUNYA) and streptavidin-PE were purchased from eBioscience. Biotin-conjugated anti-mouse IL-1R1 (JAMA-147) was purchased from Biolegend. Samples were acquired on an LSRII instrument (BD Biosciences) and data was analyzed using CellQuest Pro (BD Biosciences) or FlowJo software (Tree Star Inc.).

### Quantification of cytokine production by cytokine-stimulated effector T cells

*In vitro*-polarized Th17 or Th1 cells or *ex vivo* CD4 T cells were incubated with only the indicated recombinant cytokines at the following concentrations: IL-1β (10 ng/ml), IL-6 (20 ng/ml), IL-18 (50 ng/ml), IL-23 (10 ng/ml), TGFβ (5 ng/ml) and IL-12 (10 ng/ml), all purchased from R&D Systems. Control cultures were left without additional cytokines or plated in wells coated with anti-CD3 and supplemented with soluble anti-CD28 (5 μg/ml). Supernatants were collected after 48 hours and the production of IL-17A, IL-17F and IFNγ were detected by color development (TM-Blue; Sigma) of HRP-avidin substrate (Vector) following capture by antibodies directed against mouse IL-17A, IL-17F or IFNγ and detection by biotinylated anti-mouse IL-17A, biotinylated anti-mouse IL-17F, or biotinylated anti-mouse IFNγ, respectively (all purchased from BD Biosciences, IL-17F R&D Systems). ELISA was performed as previously described [10]. IL-22 ELISA was performed according to the manufacture's instructions (R&D Systems). The amounts of cytokine were determined from standard curves established with serial dilutions of recombinant murine IL-17A, IL-17F, IL-22 or IFNγ (R&D Systems).

### RNA isolation, cDNA synthesis and real-time PCR

T cell mRNA was extracted using TRIZOL (Invitrogen) and then treated with DNA-free (Ambion). cDNA synthesis was performed using Superscript III first-strand synthesis system (Invitrogen). Real-time PCR was performed on a Bio-Rad iCycler with primer pairs specific for cDNAs of *Il1r* and *Il18r* mRNA transcripts using SYBR GreenER qPCR supermix (Invitrogen). Primer sequences used were: *Il1r*, sense primer, 5’-TCCTGAGCCCTCGG AATG-3’, antisense primer, 5’-CGTGACGTTGCAGATCAGTTG-3’; *Il18r*, sense primer, 5’-TGGAGGATGAGGGAACGTACA-3’, antisense primer, 5’-CTTTTGGTGACATTTAA GGTCCAA-3’; *Il12rβ1*, sense primer, 5’-TACAGTTCAGGCGCCGGAT-3’, antisense primer, 5’-AGAGTTAACCTGAGGTCCGCAG-3’; *Il12rβ2*, sense primer, 5’-CCTCTTAA CAGCACGTCCTGG-3’, antisense primer, 5’-GGTCTCAGATCTCGCAGGTCA-3’; *Il23r*, sense primer, 5’-GCCAAGAAGACCATTCCCGA-3’, antisense primer, 5’-TCAGTGCT ACAATCTTCTTCAGAGGACA-3’. Reactions were run in triplicate and normalized to 18S rRNA.

### Western blotting

Viable CD4 T cells were purified on a Ficoll gradient and treated with lysis buffer (RIPA buffer; 50 mM Tris-HCl, 150 mM NaCl, 1 mM EDTA, 1% Triton X-100, 1% Sodium deoxycholate, 0.1% SDS) containing protease inhibitor mixture (Roche), 1 mM NaF (Sigma-Aldrich) and 1 mM NA_3_VO_4_ (Sigma-Aldrich). Protein samples were separated by SDS-PAGE and transferred to a PVDF membrane (Millipore). Primary antibody directed against STAT3, phospho-STAT3 (Cell signaling Technology), STAT4 (Santa Cruz) and phospho-STAT4 (Santa Cruz) and then HRP-cojugated Donkey anti-rabbit antibody (Affinity Bioreagents) were used to detect target protein by ECL detection kit (GE Healthcare).

### Small interfering RNA (siRNA)

*Stat3*-specific siRNA or Allstar negative control siRNA (Qiagen) was administrated to Th17 cells by mouse T cell nucleofector kit from AMAXA. Silencing of STAT3 in Th17 cells was verified by western blot on individual aliquots at 3 days following transfection. The remaining *Stat3*-silenced Th17 cells were then re-stimulated with indicated cytokine(s).

### Statistical analysis

Statistical significance was calculated by unpaired Student’s t test, Mann-Whitney *U* or ANOVA as appropriate, using Prism software (GraphPad; San Diego, CA). All *p* values ≤ 0.05 are considered significant, and are referred to as such in the text. Unless otherwise specified, all studies for which data are presented are representative of at least two similar studies.

## Ethics statement

All animal work was conducted at the University of Alabama at Birmingham (UAB) with the approval of the UAB Institutional Animal Care And Use Committee (IACUC). All animal facilities at UAB are under the direction of full-time veterinarians and are fully accredited by the American Association for Accreditation of Laboratory Animal Care. UAB complies with the NIH policy on animal welfare, the Animal Welfare Act and other applicable federal, state and local laws. Adequate care of the animals involved in this study was provided in accordance with the standard incorporated in The Guide to the Care and Use of Laboratory Animals, DHEW Publication No. (NIH) 78–23. Euthanasia was performed using inhalation anesthesia with isoflurane followed by swift cervical dislocation. This method is consistent with the American Veterinary Medical Association (AVMA) Guidelines for the Euthanasia of Animals.

## Results

### Th17 cell differentiation is accompanied by up-regulation of genes encoding receptors for pro-inflammatory cytokines

**Fig 1:**
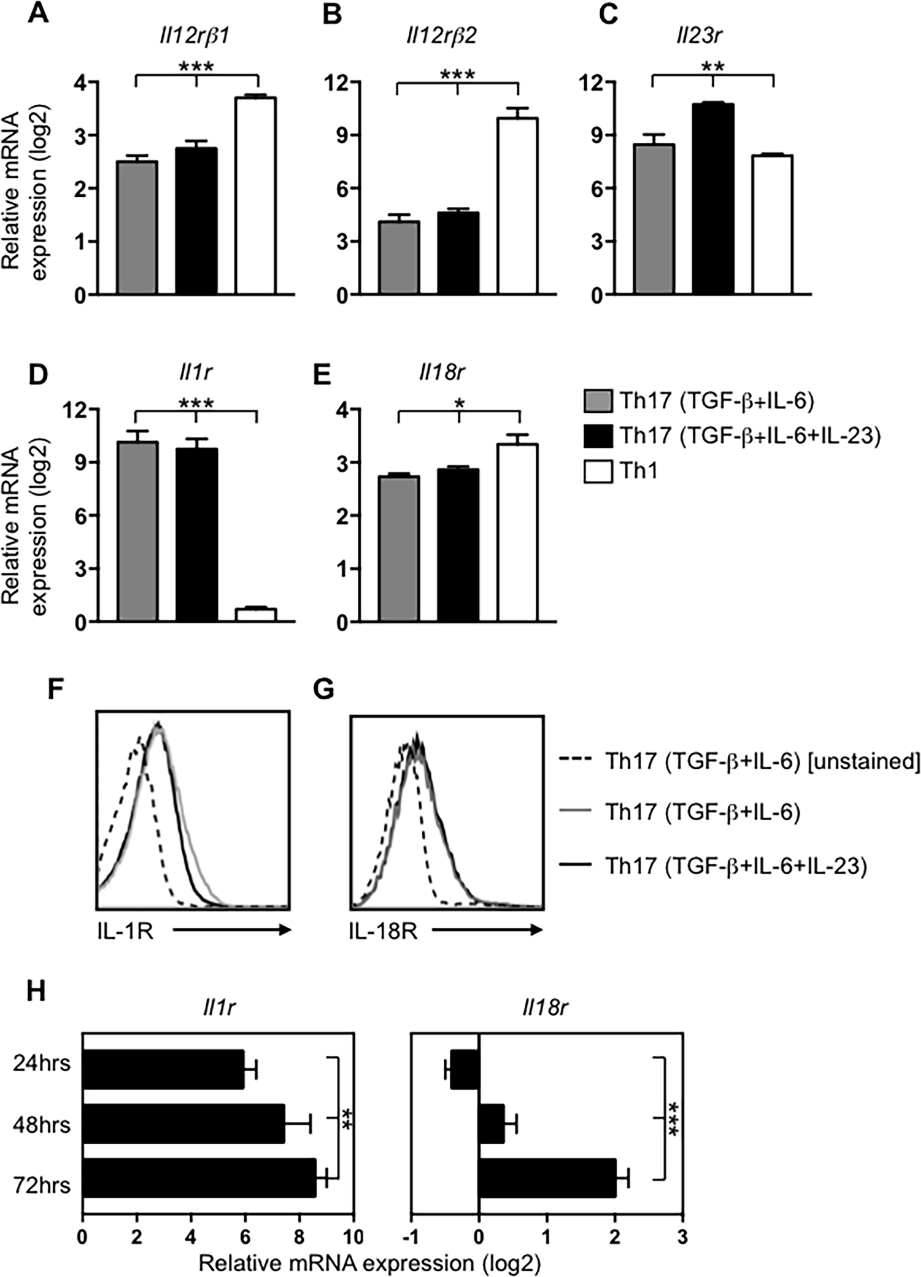
Acquisition of cytokine responsiveness by developing Th17 cells. Naïve T cells were activated in the presence of IL-12/23p40-deficient feeder cells under Th17-polarizing conditions in the presence or absence of exogenous IL-23, or under Th1-polarizing conditions. On day 5, entire cell populations were examined for expression of (A) *Il1r*, (B) *Il18r*, (C) *Il12rβ2*, (D) *Il12rβ1*, and (E) *Il23r* mRNA by real-time PCR, or examined by flow cytometry for surface expression of (F) IL-1R and (G) IL-18Rα. (H) CD4 T cells were activated under Th17-polarizing conditions with plate-bound anti-CD3 and soluble anti-CD28 and assessed after 24, 48, and 72 hours for expression of *Il1r*, and *Il18r*. PCR results are pooled from at least three independent experiments and were normalized to 18S rRNA and are expressed as fold differences (log2) relative to naïve CD4 T cells. (*p<0.05, **p<0.005, ***p<0.001).

In order to determine the potential cytokine responsiveness that is imprinted during primary Th17 cell differentiation, we first examined the expression by newly differentiated Th17 cells of mRNA transcripts encoding the receptors for multiple pro-inflammatory cytokines. In addition, we examined the effect of exogenous IL-23 on these transcripts by including IL-23 in the primary cell culture. To control the availability of IL-23, cultures were performed using IL-12/23p40-deficient (*p40*-/-) splenic feeder cells. Naïve OT-II TCR transgenic CD4 T cells were cultured for 5 days under Th1- or Th17-polarizing conditions, the latter in the presence or absence of IL-23. *Il1r*, *Il18r*, and *Il12rβ2* were all up-regulated on Th17 cells but the transcript levels were unaffected by the addition of IL-23 (**Fig 1A–1C**). In contrast, and consistent with published results [10, 24], *Il12rβ1* and *Il23r* were up-regulated on Th17 cells and the expression of both increased in the presence of IL-23 (**Fig 1D and 1E**). Importantly, whereas both Th1 and Th17 cells expressed *Il18r*, *Il1r* was selectively induced on Th17 cells suggesting that of these two lineages, IL-1-dependent functional enhancement is unique to the Th17 lineage. In addition to mRNA expression, we confirmed surface expression of both IL-1R and IL-18R by Th17 cells after 5 days of polarization (Fig 1F and 1G). Kinetic analysis of *Il1r* and *Il18r* up-regulation on Th17 cells revealed that *Il1r* is highly up-regulated as early as 24 hours post-stimulation and (**Fig 1F**, left) but, that *Il18r* is up-regulated later during development, appreciably detectable by 72 hours after initial polarization (**Fig 1F**, right). Thus, whereas signaling via the IL-1R might at least contribute to primary Th17 development, IL-18R signaling appears to be dispensable for Th17 development, but is gradually established coincident with lineage commitment. These data point to a possible role for IL-18R in lineage-committed Th17 cells, similar to its role in TCR-independent IFNγ production by committed Th1 cells [25, 26].

### IL-23 acts synergistically with IL-1β or IL-18, to promote TCR-independent cytokine production by Th17 cells

**Fig 2:**
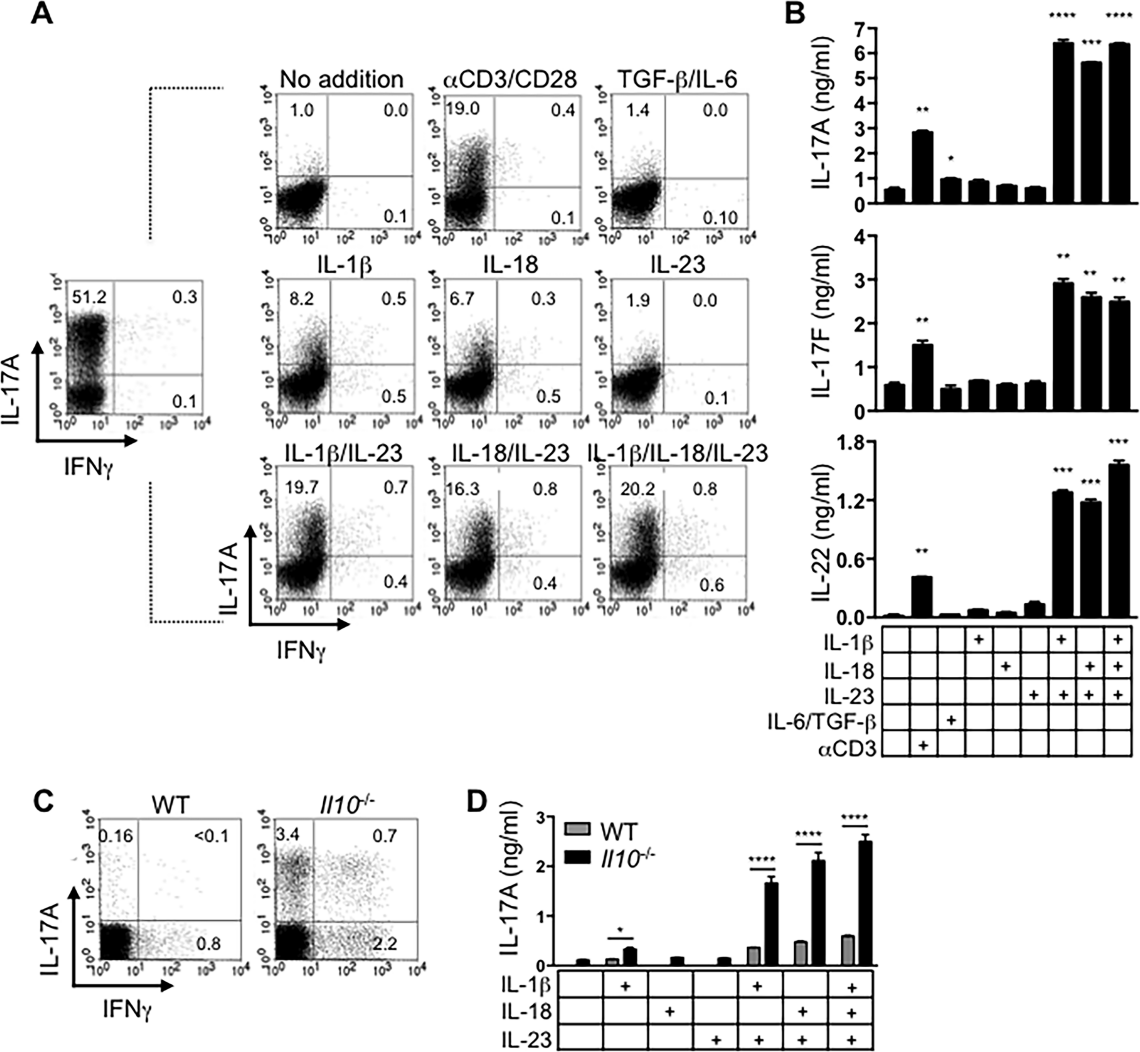
IL-23 acts synergistically with IL-1β or IL-18 to induce production of Th17-associated cytokines by committed Th17 cells. (A) Naïve CD4 T cells were cultured with irradiated *p40-/-* splenic feeder cells under Th17-polarizing condition for 3 rounds of 7 days each. A portion of the cells was then stimulated with PMA/ionomycin in the presence of monensin and stained intracellularly for IL-17A and IFNγ to confirm their differentiation into Th17 cells (left). Of the remaining cells, 1x106 cells/well were re-stimulated with indicated cytokine(s) for 12 h with monensin added for the final 5 hours, and examined for intracellular expression of IL-17A and IFNγ. (B) 2x105 highly polarized FACS-sorted Thy1.1+ (IL-17F+) cells were either left unstimulated, re-stimulated with anti-CD3 and anti-CD28, or with the indicated cytokines for 48 hours. ELISA was performed to quantify IL-17A, IL-17F, and IL-22 in culture supernatants. (C) CD4 T cells were isolated from MLNs of either WT (grey bars) or colitic IL-10-deficient mice (black bars) and stimulated with PMA plus ionomycin for 5 h in the presence of monensin before being stained intracellularly for IL-17A and IFNγ. (D) Total MLN CD4 T cells (5x105 cells/well) from WT or *Il10-/-* CD4 T cells were stimulated with indicated cytokine(s) for 48 hours and the concentration of IL-17A in culture supernatants was determined by ELISA. Error bars represent means ± s.d. of triplicate determinations. Data are representative of at least three independent experiments (*p<0.05, **p<0.01, **p<0.001 and **p<0.0001).

Based on the foregoing data, we then asked whether fully committed Th17 cells, through up-regulation of the aforementioned receptors, could be activated to express Th17 signature cytokines in response to the corresponding cytokines and in the absence of further TCR ligation. Considering the reported ability to IL-23 to enhance Th17 effector responses, we also examined the possible involvement of IL-23 signaling in this process. Thus, as in Fig 1, we utilized *p40*-/- splenic feeder cells in all cultures. Naïve T cells were subjected to three rounds of activation under Th17-polarizing conditions to generate highly differentiated Th17 cells as evidenced by the detection of a high frequency of IL-17-positive (51% of viable CD4 T cells) and absence of IFNγ-positive cells after PMA and ionomycin stimulation (**Fig 2A**, left). The total CD4 T cell population was then re-stimulated with the indicated cytokine combinations for 12 hours and stained for intracellular IL-17A and IFNγ. As expected, unstimulated controls or TGFβ/IL-6-stimulated cells did not readily secrete IL-17 whereas activation of the TCR-CD3 complex induced IL-17 secretion (**Fig 2A**, upper panel). Re-stimulation with only IL-1β or IL-18, but not IL-23, induced robust expression of IL-17A (8.2% and 6.7% respectively), with very little IFNγ (≤1% in each case) (**Fig 2A** middle panel). Interestingly, while IL-23 alone failed to induce comparable levels of IL-17A, the combination of IL-1β and IL-23, or IL-18 and IL-23 induced elevated expression of IL-17A (20% and 16%, respectively) that resembled the cytokine production in response to anti-CD3 re-stimulation. The combination of IL-1β, IL-18, and IL-23 did not appear to have an additive effect on the IL-17A production (**Fig 2A**, lower panel). Furthermore, not only were the frequencies of IL-17+ cells elevated when stimulated with IL-23 plus IL-1β or IL-18, so was the fluorescence intensity of the cytokine staining. Thus IL-23 synergized with IL-1β or IL-18 to promote enhanced secretion of IL-17A relative to what was induced by these cytokines individually.

To quantify the production of Th17 cytokines in response to cytokine re-stimulation, we utilized CD4 T cells from IL-17F/Thy1.1 knock-in (IL-17F KI) reporter mice that permit isolation of IL-17F-expressing cells via Thy1.1 reporter molecule [10]. CD4+Thy1.1+ cells were isolated from Th17 cultures and either left untreated (no addition), stimulated with anti-CD3 and anti-CD28, or re-stimulated with the indicated cytokine combinations for 48 hours. Supernatants were then examined for IL-17A, IL-17F, and IL-22 (**Fig 2B**). Consistent with the intracellular cytokine staining, IL-1β, IL-18, and IL-23 induced minimal-low level production of IL-17A, but this increased ~6-12-fold when either stimulant was paired with IL-23, or when all 3 cytokines were added to the same culture (**Fig 2B**). A similar trend was observed when we measured the levels of IL-17F and IL-22. Interestingly, in the absence of TCR stimulation, TGFβ and IL-6 failed to induce robust levels of Th17-associated cytokines. Despite promoting similar frequencies of IL-17A-producing CD4 T cells, activation the TCR-CD3 complex in the absence of cytokine stimulation induced markedly lower amounts of all 3 cytokines measured. This might be due to the differential kinetics of cytokine production in response to TCR versus cytokine activation, where TCR stimulation induces rapid, early cytokine release, while cytokine stimulation results in a more sustained production and accumulation of cytokines during the 48-hour period. Notably, IL-23 signaling has been reported to induce expression of IL-22 [27] but under our experimental conditions, in the absence of TCR stimulation, IL-23 alone induced near background levels of IL-22. However, IL-23 in the presence of IL-1β or IL-18 induced a large quantity of IL-22. Taken together, these data demonstrate that *in vitro*-differentiated Th17 cells can be induced to express Th17 signature cytokines in a TCR-independent manner via the synergistic effects of IL-23 and the IL-1 family cytokines IL-1β or IL-18.

### IL-23 in combination with IL-1β, IL-18 or both induces IL-17A production by *in vivo* generated Th17 cells independently of TCR stimulation

To determine whether the TCR-independent induction of Th17 cytokines occurs in *in vivo* Th17 effector/memory cells, we repeated the experiment using CD4 T cells from mesenteric lymph nodes (MLN) of colitic IL-10-deficient mice. Indicative of ongoing intestinal inflammation, IFNγ- and IL-17A-competent cells were present in MLN of the colitic mice, but importantly, not in the healthy WT control mice **(Fig 2C)**. Similar to *in vitro*-differentiated Th17 cells, colitogenic CD4 T cells produced significant levels of IL-17A in response to stimulation with IL-1β plus IL-23 and IL-18 plus IL-23 whereas WT cells did not **(Fig 2D)**. Thus, although our cytokine quantification was done using bulk IL-10-deficient CD4 T cells, a small fraction (~4%) of which were IL-17+ by intracellular staining, the limited production of IL-17A by WT CD4 T cells, the high concentration of IL-17A relative to the *in vitro*-derived Th17 cells, and the fact that no Th17-polarizing cytokines were included in our brief stimulation cultures, suggest that the observed cytokine response is predominantly, if not completely, due to the IL-10-deficient Th17 effectors. Thus, similar to the *in vitro*-generated Th17 effectors, inflammatory Th17 cells retain the capacity to produce their signature cytokines in response to cytokine stimulation with IL-23 in the presence of IL-1, IL-18 or both. This pathway likely enhances the output of Th17 cytokines by these cells that drive the ongoing inflammation in the absence of IL-10 production.

### IL-23 signaling during primary Th17 differentiation enhances subsequent TCR-independent cytokine responses

**Fig 3:**
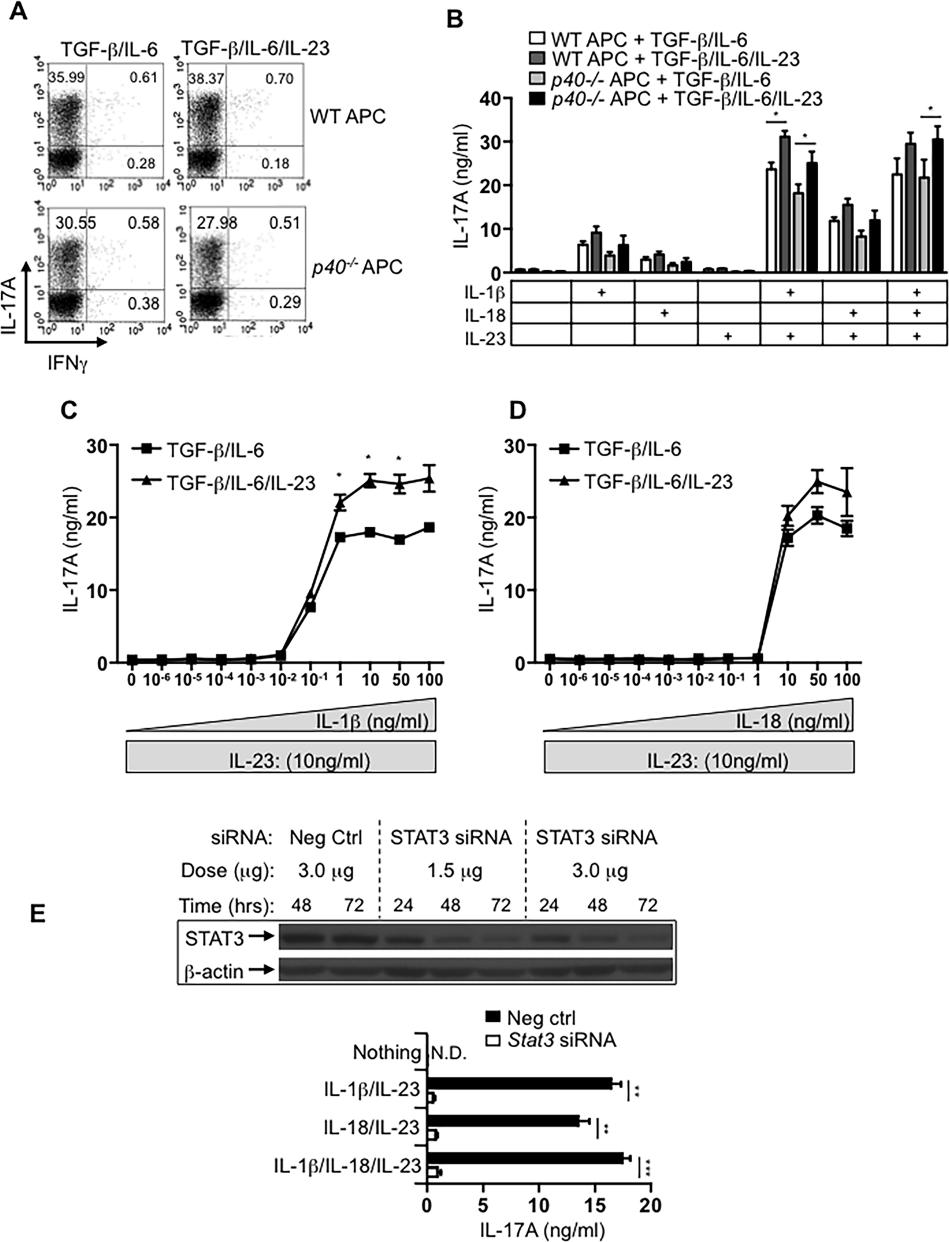
IL-23 signaling during Th17 differentiation enhances TCR-independent production of IL-17A by committed Th17 effectors in STAT3-dependent manner. (A) Naïve CD4 T cells were cultured for 3 rounds under Th17 polarizing conditions with WT or *p40-/-* splenic feeder cells in the absence or presence of exogenous IL-23. Cells were stained intracellularly for IL-17A and IFNγ after PMA/ionomycin activation for 5 h in the presence of monensin. (B) CD4 T cells from the cultures in (A) were purified and the re-stimulated at a density of 1x106 IL-17A+ cells/ml (based on the frequencies shown in (A) with indicated cytokine(s) for 48 hours. ELISA was performed to measure IL-17A concentration in culture supernatants. (C) Th17 cells, polarized as in (A), were re-stimulated with titrated doses of IL-1β or IL-18 and a constant dose of IL-23 for 48 hours. ELISA was performed to measure IL-17A concentration of the culture supernatant. (E) Th17 cells polarized for 3 rounds were purified and Stat3-specific siRNA (1.5 or 3.0 μg) or Allstar negative control (3.0 μg) was administered. Aliquots of cultured cells were examined daily by western blot for expression of STAT3. After 72 hours, Th17 cells that had been treated with 3.0 μg of STAT3 siRNA were left untreated or were treated for 48 hours with the indicated cytokines and the concentration of IL-17A in culture supernatants was determined by ELISA. Error bars on all graphs represent means ± s.d. of triplicate determinations. All data are representative of at least three independent experiments (*p<0.05, **p<0.01 and **p<0.001).

The cytokine IL-23 is largely dispensable for the induction of Th17 cells but is known to promote the stability (preservation of the Th17 program)–as well as pathogenicity–(pro-inflammatory potential) of fully differentiated Th17 cells. As TCR-independent activation could be one mechanism to promote sustained Th17 functions in certain settings, we wondered whether IL-23 stimulation during development, had any impact on the subsequent TCR-independent effector program of Th17 cells. To test this, we utilized a system in which our APC were unable to produce IL-12 or IL-23. Naïve OT-II TCR transgenic CD4 T cells were cultured with WT or *p40*-/- irradiated feeder cells under Th17-polarizing conditions for three rounds, in the absence or presence of exogenous IL-23. In the presence of WT feeder cells, addition of IL-23 resulted in a modest increase in the frequency of IL-17+ cells (36% in the absence of IL-23 versus 38% in the presence of IL-23). The same was true of Th17 cells cultured with *p40*-/- feeder cells where inclusion of IL-23 produced a slight increase in IL-17+ cells (30% without IL-23 versus 34% with IL-23) (**Fig 3A**). Under both conditions (TGFβ/IL-6 and TGFβ/IL-6/IL-23), Th17 differentiation on WT feeder cells resulted in slightly higher frequencies of Th17 cells compared to those differentiated on *p40*-/- feeder cells perhaps indicative of a minor contribution for feeder cell derived IL-23 that becomes more apparent in long-term cultures of Th17 cells. We then stimulated equal numbers (based on the intracellular cytokine staining) of IL-17-competent CD4 T cells with the cytokine combinations shown. Similar to our earlier results, IL-23 in combination with IL-1β, IL-18 or both resulted in the production of IL-17A production by Th17 cells differentiated in the absence of IL-23. More importantly, the presence of exogenous IL-23 during polarization resulted in a statistically significant increase in the amount of IL-17A secreted by cells stimulated with IL-23 plus IL-1β, IL-23 plus IL-18, or all three cytokines (**Fig 3B**). Consistent with the observations detailed in Fig 3A, differentiation in the presence of IL-23 enhanced the subsequent TCR-independent production of IL-17A in response to IL-23 and IL-1β or IL-18. These data identify another role for IL-23 during Th17 commitment; to increase the propensity of cells to rapidly secrete high levels of IL-17A even in the absence of further direct antigenic stimulation.

As shown in Fig 1A, *Il1r* is more rapidly up-regulated and more highly expressed on Th17 cells than *Il18r*. Accordingly, in the presence of IL-23, IL-1β consistently induced higher levels of IL-17A, although the magnitude of this difference varied somewhat between experiments, and despite our use of 5-fold higher concentrations of IL-18 based on published information [26]. These data suggested that IL-1β is more potent than IL-18 in its ability to induce TCR-independent Th17 responses. We therefore sought to determine the exact sensitivity of Th17 cells to these two cytokines. Thus, Th17 cells were cultured for three rounds in the presence or absence of IL-23 and then re-stimulated with a fixed dose of IL-23 in the presence of titrated doses of IL-1β or IL-18 (**Fig 3C and 3D**). We detected IL-17A in response to stimulation with as little as 100 pg/ml of IL-1β and the cytokine output approached maximal levels in response to 1 ng/ml of IL-1β (**Fig 3C**). In contrast, the same concentration of IL-18 was insufficient to induce IL-17A production by Th17 cells (**Fig 3D**). Instead, near-maximum IL-17A was detected in response to 10 ng/ml IL-18, with the output peaking in response to 50 ng/ml. Altogether, these data indicate Th17 cells rapidly acquire enhanced responsiveness to IL-1β, which serves as a dominant inducer of IL-17 production from committed Th17 effectors.

STAT3 signaling is critical for IL-17 induction and for sustained expression of RORγt in developing Th17 cells [28–30]. Thus, we sought to confirm the role of STAT3 in the IL-23-mediated, TCR-independent recall responses of fully committed Th17 cells. We utilized STAT3-small interfering RNA (siRNA) instead of STAT3-deficient CD4 T cells since siRNA allowed us to selectively inhibit *Stat3* after Th17 differentiation. Th17 cells were generated as before by culturing for 3 rounds. The total cell population was then transfected with *Stat3* siRNA for 3 days and an aliquot was tested daily by western blot to verify suppression of STAT3 (Fig 3E, upper panel). STAT3-suppressed cells were then activated or not with the indicated cytokines for 48 hours (**Fig 3E**). Suppression of STAT3 significantly impeded the production of IL-17 by cells stimulated with IL-1β and IL-23, IL-18 and IL-23, or all three cytokines combined **(Fig 3E**, lower panel) demonstrating that STAT3 signaling in response to IL-23 is responsible for the production of IL-17A via the TCR-independent pathway. Therefore, IL-23-dependent activation of STAT3 is necessary for the TCR-independent production of IL-17 in response to IL-23 and IL-1 family cytokines.

### Th17 cells transition to IFNγ-producers in response to IL-12 stimulation in the absence of TCR ligation

**Fig 4:**
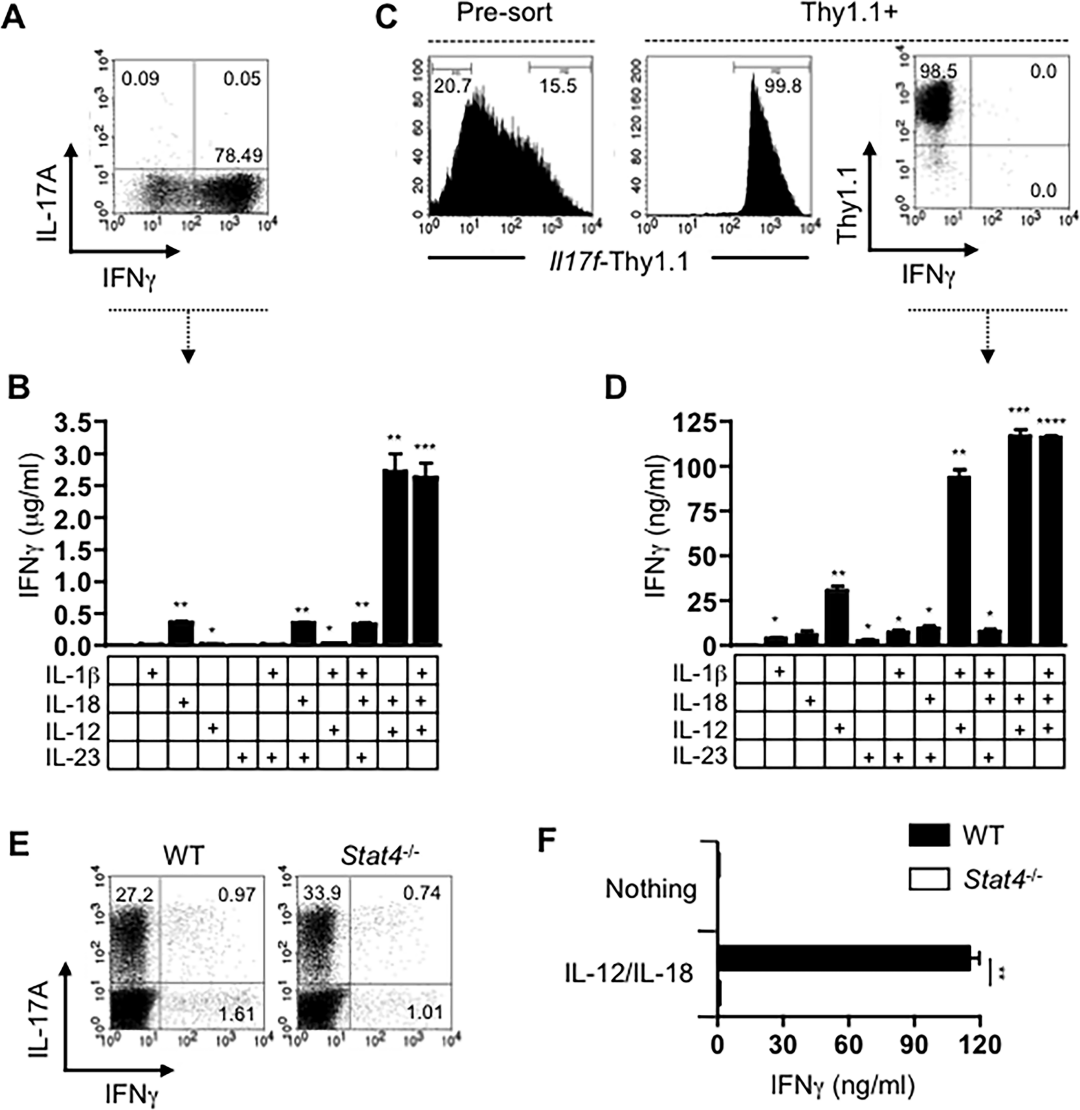
IL-12 synergizes with IL-1β or IL-18 to induce the transition of Th17 cells to IFNγ-expressing progeny independent of TCR stimulation. Bulk Th1 cells (A) were activated as shown for 48 hours and the concentration of IFNγ in culture supernatants was determined by ELISA (B). Naïve CD4 T cells from *Il17f/Thy1.1* reporter mice were differentiated into Th17 cells and the Thy1.1hi fraction was enriched by FACS (C). Cells were then treated with indicated cytokine combination for 48 hours and ELISA was performed to measure the concentration of IFNγ in culture supernatants (D). To determine the role of STAT-4, naïve CD4 T cells from WT or *Stat4-/-* mice were cultured under Th17 polarizing conditions and their differentiation confirmed by intracellular cytokine staining (E). The number of IL-17A+ T cells was normalized based on intracellular cytokine staining then re-stimulated with indicated cytokine(s) for 48 hours and ELISA was performed to measure IFNγ concentration. Error bars represent means ± s.d. of triplicate determinations. Flow cytometry plots are gated on CD4+ cells and the numbers represent the percentages of cells in each quadrant. Data are representative of at least three independent experiments (*p<0.05, **p<0.01, **p<0.001 and **p<0.0001).

We previously demonstrated that in response to IL-12, Th17 cells down-regulate expression of IL-17 and transition to IFNγ-producing cells in a STAT4-dependent manner [10]. We next examined whether this plasticity can be activated in the absence of TCR signaling and whether there is a role for IL-1β and/or IL-18 in this process. As a control population, we utilized bulk Th1 cells (**Fig 4A**), which are known to produce IFNγ in response to stimulation with IL-12 plus IL-18 [25, 26], and as expected, this combination of cytokines induced high-level expression of IFNγ (**Fig 4B**). Consistent with the lack of *Il1r* expression by Th1 cells (Fig 1A), the inclusion of IL-1β had no impact on their expression of IFNγ. To generate Th17 cells, we utilized naïve cells purified from the previously utilized IL-17F-Thy1.1 reporter mouse in which activation of the *Il17* locus results in surface expression of Thy1.1. Th17 cells were differentiated over a 7-day period and Thy1.1^hi^ cells were enriched by FACS sorting to a purity of >98%. Importantly, these cells did not produce IFNγ when re-activated with PMA and ionomycin (**Fig 4C**). Purified Th17 cells were stimulated with the cytokine or combinations of cytokines shown and the amount of IFNγ produced was determined by ELISA. As with Th1 cells, IL-1β, IL-18, or IL-23 alone, or the combination of IL-23 and either of these 2 cytokines, did not induce IFNγ production by Th17 cells (**Fig 4D**). However, IL-12 independently induced low, but detectable levels of IFNγ from Th17 cells (**Fig 4D**). Stimulation of Th17 cells with IL-12 and IL-1β, or IL-12 and IL-18 resulted in approximate 3- and 4-fold increases respectively in IFNγ production compared to stimulation with IL-12 only. Thus, like Th1 cells, Th17 cells express IFNγ when re-stimulated with IL-12 and IL-18, but unlike Th1 cells, which do not express IL-1R, Th17 cells express IFNγ when activated with the combination of IL-12 in the presence of IL-1β. Importantly, whereas in the presence of IL-23, IL-1β was more potent than IL-18 at inducing Th17-related cytokines from Th17 cells, in the presence of IL-12, IL-18 constantly induced elevated levels of IFNγ from Th17 cells. Admittedly, Th1 cells produced approximately 25 times more IFNγ than Th17 cells when stimulated with IL-12 and IL-18, likely due to the differences between a completely remodeled and transcriptionally active *Ifng* locus in Th1 cells versus a “poised” *Ifng* locus in Th17 cells that is further remodeled in response to IL-12 [31]. Altogether, these data demonstrate, that in the absence of TCR signaling, Th17 cells can still transition of IFNγ-expressing progeny through the synergistic effects of IL-12 and IL-1 family cytokines IL-1β or IL-18.

Finally, to confirm the involvement of IL-12R signaling, we examined the requirement for STAT4 in the IL-12-induced up-regulation of IFNγ by Th17 cells. We asked whether STAT4 signaling was critical for the induction of IFNγ production by Th17 cells upon stimulation with IL-12 and IL-18. Th17 cells differentiated from WT as well as *Stat4*-/- CD4 T cells that importantly, show no defect in Th17 development (**Fig 4E**). We then stimulated the bulk T cell populations with IL-12 and IL-18 for 48 hours before quantifying the amount of IFNγ in the culture supernatants. As expected, IL-12 synergized with IL-12 and IL-18 to induce IFNγ production by WT Th17 cells but *Stat4*-/- cells failed to up-regulate IFNγ when similarly activated. Our inability to enrich Th17 cells from non-reporter transgenic mice makes it impossible to conclusively to ascertain that the cells identified as the IL-17-producers by FACS are the sole source of the STAT4-dependent IFNγ detected. However, the intracellular staining showing that the precursors are largely IFNγ-negative (**Fig 4E**), coupled with the comparable concentrations of IFNγ detected by purified Th17 cells that were similarly stimulated (**Fig 4D**), strongly support this and thus suggest that the TCR independent transition of Th17 cells to IFNγ-producing cells is STAT4-dependent. Taken together, these data demonstrate that developing Th17 cells become imprinted with the ability to modify their phenotype to resemble Th1 cells in response to IL-12 stimulation and that this effect is amplified by the presence of IL-1 or IL-18.

## Discussion

In this report, we have dissected yet another parallel between the Th1 and Th17 effector programs–TCR-independent effector cytokine production in response to synergistic activation by cytokines of the IL-12 and IL-1 family cytokines. Specifically, IL-23, in combination with IL-1, or to a lesser extent, IL-18, induced Th17-associated cytokine production by *in vitro*-polarized as well as *in vivo*-derived Th17 cells. Moreover, stimulation with IL-23 during Th17 differentiation enhanced this effect, likely due, at least in part, to increased induction of *Il23r* in the presence of exogenous IL-23 [32]. Thus, our findings demonstrate yet another role for the IL-23-IL23R pathway in terminally differentiated Th17 cells, that is, to facilitate optimal effector cytokine production in response to antigens that activate innate immune cell production of IL-23 and IL-1 family cytokines. This function of IL-23 is contingent upon its induction of STAT3 hence the effect was completely lost when expression of *Stat3* was suppressed. Our findings are thus consistent with an earlier study demonstrating that effector T cells of all lineages can be induced to express their signature molecules provided they are simultaneously exposed to IL-1 family cytokines and a STAT activator [18]. This production of effector cytokines in response to the secreted products of innate immune cells likely represents an essential mechanism for recall Th17 responses *in vivo*, in which IL-23 has been implicated.

While antigenic stimulation is necessary for initial Th17 development, effector/memory T cells persist *in vivo*. This is particularly true of the intestinal lamina propria where Th17 cells that differentiate in response to commensal antigens persist in the steady state in specific pathogen-free mice [33, 34]. Thus, our findings may represent an avenue for bystander effector activity of gut-resident Th17 cells in response to oral infection where the release of innate pro-inflammatory IL-23 along with IL-1 and/or IL-18 can rapidly initiate effector cytokine production from memory Th17 cells, avoiding any possible delay that might be associated with differentiation of new effector cells.

Like Th1 cells, Th17 cells up-regulate the IL-18R during antigen-induced, TGFβ-and IL-6-dependent differentiation and our data recapitulates previous findings that developing Th17 cells uniquely and rapidly express *Il1r*, paving the way for the reported enhancement of Th17 differentiation by IL-1 [35, 36]. Despite some earlier discrepancies, it is now widely accepted that neither IL-1 nor IL-18 is required for minimal Th17 differentiation [37]. Hence, based on the data presented herein, it appears that the induction of these pathways predominantly represents a provision for subsequent TCR-independent Th17 function, for example, in Th17 memory responses. In addition, we noticed substantial differences in both the kinetics of up-regulation and the magnitude of expression of *Il1r* relative to *Il18r*, in that *Il1r* was up-regulated much earlier and sustained at higher levels than *Il18r*. Consistent with this, the combination of IL-23 and IL-1 consistently induced higher levels of Th17-associated cytokines than the combination of IL-23 and IL-18 although the magnitude of this difference varied slightly between experiments. In addition, the sensitivity of differentiated Th17 effectors to IL-1β was vastly superior to that of IL-18 such that greater than 1 log-fold higher doses of IL-18 were required to achieve the same response as the normal dose of IL-1β utilized throughout the study. This might explain why a previous report did not detect any induction of IL-17A in response to stimulation with IL-23 and IL-18 [18]. Therefore, although IL-18 fairly potently supports TCR-independent production of IL-17 in the presence of IL-23, IL-1β may be the dominant cofactor for this process.

In addition to the production of lineage-associated cytokines, Th17 cells acquired the capacity to transition to IFNγ-producing progeny in response to the stimulation with IL-12 in the absence of TCR ligation. Similar to our previous findings [10], this transition was dependent on IL-12-induced STAT4 signaling. This represents a slight departure from the situation observed with Th17 cells where, by itself, IL-23 was insufficient to induce IL-17 production above background levels. This points to differential susceptibility to STAT signaling at the *Ifng* and *Il17* loci; with the *Ifng* locus being acutely responsive to isolated STAT4 signaling. Indeed analysis of the chromatin landscape in Th17 cells revealed a number of transcriptionally permissive histone modifications at the *Ifng* locus in Th17 precursors, such that these cells are always poised to secrete IFNγ which is enhanced by rapid remodeling in response to IL-12 stimulation [31].

Importantly, the IL-12 induced production of IFNγ was amplified by the presence of IL-1β or IL-18. The combination of IL-12 and IL-18 is known to induce Th1, as well as Tc1 effector function in a non-antigen-specific manner [25, 26, 38], but our study provides evidence that, in the presence of IL-12, IL-1β can actually support IFNγ production by cells expressing high levels of the IL-1R. The delayed up-regulation of *Il18r* relative to *Il1r* on Th17 cells suggests that unlike IL-1R, which as previously mentioned has been implicated in enhanced Th17 differentiation, the necessity of IL-18R signaling largely lies beyond antigen-driven Th17 differentiation. In fact, whereas IL-1β plus IL-23 induced more IL-17A than IL-18 plus IL-23, IL-12 and IL-18 consistently induced slightly more IFNγ production by Th17 cells than the combination of IL-12 and IL-1β. Therefore, as with bona fide Th1 cells, IL-12 responsiveness by Th17 cells favors the IL-18 co-dependent induction of IFNγ production in response to stimuli that promote production of IL-12 by innate immune cells.

Altogether, we have demonstrated that Th17 cell acquisition of responsiveness to both IL-12 and IL-23 allows for subsequent toggling of the fate of these cells in response to innate cell activation. Indeed, differential expression of IL-12 versus IL-23 by innate cells responding to specific antigens has been reported [39, 40]. This likely enables Th17 cells to function as multi-potent effectors, able to respond in a bystander fashion to either IL-12 or IL-23, with IL-1β and IL-18 serving as essential cofactors for both processes.
